# Paralysis of the Pneumogastric Nerve

**Published:** 1867-07

**Authors:** 


### *Paralysis of the Pneumogastric Nerve.*

Prof. Wolff, of Berlin, narrates the following interesting case, as an appendix to a lengthy paper on defective innervation of the heart. The paper is published in the *Deutsche Klinik*, of October 23, 1866.

General von S., a friend of the deceased, aged 68, otherwise robust, had for about a year complained of an annoying pain in the occiput, periodically occurring, and which required the head to be supported; when, on a sudden, he was seized with symptoms which could only be accounted for by the assumption of a beginning paralysis of the par vagum. The three functions over which these nerves preside were simultaneously and equally affected; the appetite gave way entirely, and during the two weeks that illness lasted, could not by any means whatever be restored. With great reluctance, the patient contrived, in submission to medical order, to swallow a small quantity of food or beverage. The respiration slackened more and more; and on one of his last days, there were counted forty-four seconds between two respirations. *Pari pasu* declined the movements of the heart. As its impulse and sounds grew feebler, so the pulse vanished in the extremities, and at length could no more be felt in the crural and radial arteries. During the two last days of life, it was by the stethoscope only that a faint vibration of the heart could be perceived. Along with the pulse went also the warmth of the extremities. ‘ However, gangrene of the feet did not ensue; the occurrence of which I had occasion to observe (together with Drs. Romberg and Riese) about the same time in a patient who succumbed to fatty disease of the heart, and in whose case the pulse and temperature had likewise fallen off in the low extremities during the last days of his life.’ Death, when ensued at the commencement of the third week of illnes, while the patient remained conscious to his last breath, was in the true sense of the word an ‘ extinction ’ of life. The criminal cavity was not allowed to be opened.”—*British Medical Journal.*

